# Cerebral venous sinus thrombosis and dural arteriovenous fistula associated with protein S deficiency: a case series study

**DOI:** 10.1186/s12883-022-02693-3

**Published:** 2022-05-02

**Authors:** Hui Liang, Congjie Xu, Jiyi Xu

**Affiliations:** 1 Department of Neurology, Hainan General Hospital, Hainan Affiliated Hospital of Hainan Medical University, Supported by Hainan Province Clinical Medical Center, Haikou, China; 2 Department of Urology, Hainan General Hospital, Hainan Affiliated Hospital of Hainan Medical University, Haikou, China; 3 The National Clinical Research Center for Mental Disorders & Beijing Key Laboratory of Mental Disorders, Beijing Anding Hospital, Capital Medical University, Beijing, China; 4 Advanced Innovation Center for Human Brain Protection, Capital Medical University, Beijing, China

**Keywords:** Cerebral venous sinus thrombosis, Dural arteriovenous fistula, Protein S deficiency, Clinical characteristics

## Abstract

**Objective:**

To describe the characteristics of patients with cerebral venous sinus thrombosis (CVST) and dural arteriovenous fistula (AVF) associated with protein S (PS) deficiency.

**Methods:**

We conducted a search of medical records in Hainan General Hospital from January 2000 to December 2020 for coexistence of CVST and dural AVF associated with PS deficiency and searched PubMed、Embase and Chinese biomedical databases (CBM) for all literature describing CVST and dural AVF with PS. We analyzed clinical characteristics, location, sequence of CVST and dural AVF, level of PS, therapeutic methods and prognosis.

**Results:**

We presented 1 patient in our hospital’s database combined CVST and dural AVF associated with PS, plus 5 cases reported in literature. The most common symptoms were headache, generalized seizure, disturbance of consciousness. The most frequent location of CVST was at internal cerebral vein, while transverse sinus, sigmoid sinus, parietal region in dural AVF. Two patients developed dural AVF several months or years after CVST. Clinical characteristics and level of PS were summarized.

**Conclusion:**

These findings alert physicians to consider PS deficiency in patients who suffer from CVST, especially those combined with dural AVF.

## Introduction

Cerebral venous sinus thrombosis (CVST) is a kind of venous system disease which is characterized by thrombosis of cortical veins, dural sinus, proximal internal jugular veins [1]. The clinical symptoms, affected by the site of thrombosis, range of lesions, the degree of cerebral tissue damage, are complex and varied, which bring a huge difficulty to clinical diagnosis and treatment [2]. Furthermore, compared with arterial thrombosis system, the incidence of CVST is lower, especially when it is combined with dural arteriovenous fistula (AVF) [3].

Protein S (PS) is a potent anticoagulant that regulates thrombin formation and is a vitamin K-dependent glycoprotein which is primarily synthesized in the liver [4]. A deficiency in this protein or decreased activity can cause a hypercoagulable state with increased risk of thrombolism. Of 71 PS deficiency members from 12 Dutch pedigrees, 74, 72, 38% of the individuals sustained deep vein thrombosis, superficial thrombophlebitis and pulmonary emboli, respectively [5]. Axillary and mesenteric vein thrombosis [5, 6] as well as arterial thrombosis [7, 8] has been rarely reported. Furthermore, CVST is a rare manifestation of PS deficiency. However, CVST together with dural AVF induced by PS deficiency is even rare on clinical.

Here we report a case, with CVST complicated with dural AVF induced by PS deficiency, from our hospital database and 5 additional cases retrieved from the available medical literature. The detailed clinical characteristics, location, sequence of CVST and dural AVF, level of protein S, therapy, outcomes are provided.

## Materials and methods

### Design

Collect informations of patients with combination of CVST and dural AVF related with deficiency with PS via our database and public database (PubMed, Embase and Chinese biomedical databases) and then summarize clinical characteristics of those patients.

### Retrospective study of our database

We searched all medical records at Hainan General Hospital (HGH) from January 2000 to December 2020 for International Classification of Diseases (ICD) -9 and ICD-10 diagnostic codes for coexistence of CVST and dural AVF. We retrospectively re-evaluated the diagnoses based on clinical presentations and neuroradiological examinations, then followed up with patients by telephone.

### Search of the literature

**Fig. 1:**
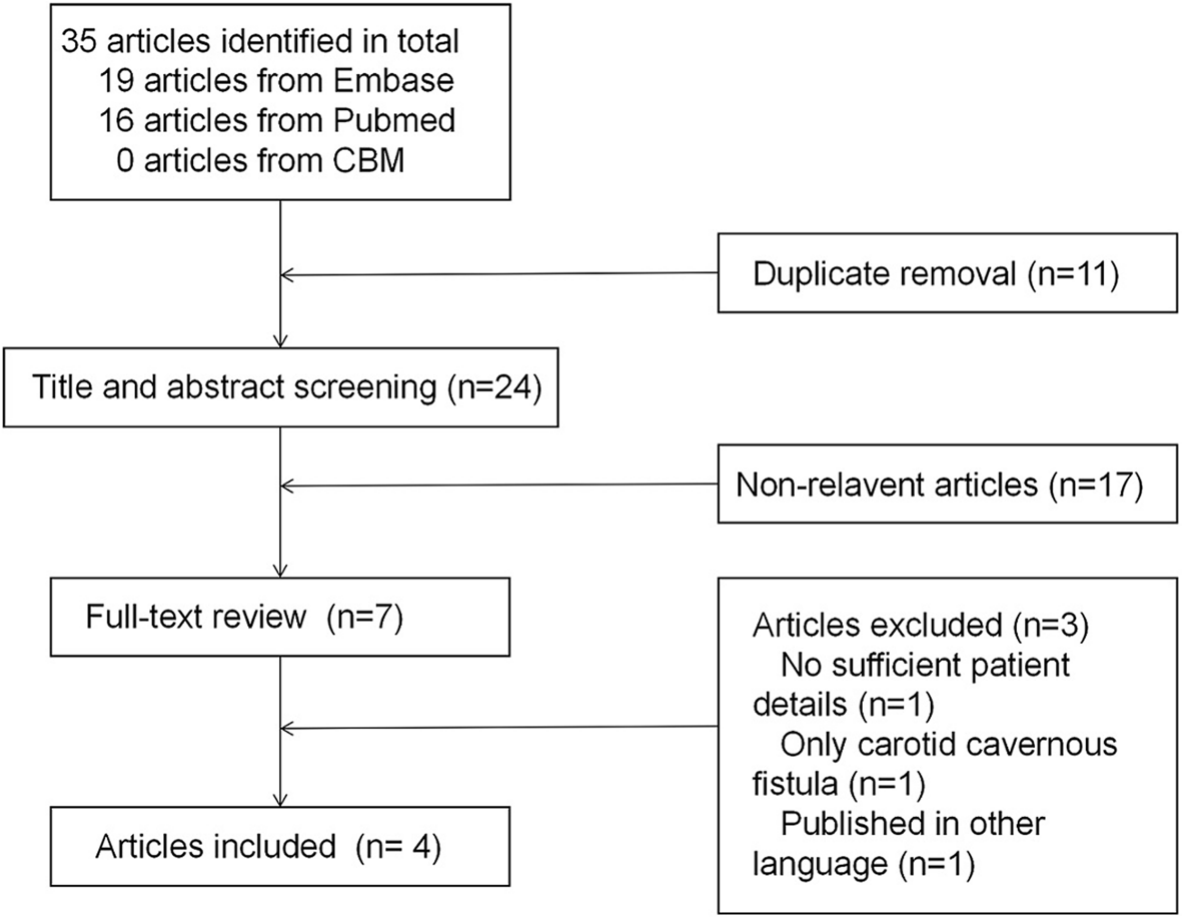
Flow chart of article retrieval

The PubMed, Embase and Chinese biomedical databases (CBM) were used to searched for reports of CVST and dural AVF associated with PS deficiency. The search of electronic databases combined disease-specific terms (cerebral venous sinus thrombosis or intracranial venous sinus thrombosis or sinus thrombosis) and (dural arteriovenous fistula or arteriovenous fistula) with PS. A total of 35 articles were identified. After removing duplicate articles, the 24 remaining articles were screened by title and abstract. Only 7 full-text articles were assessed for eligibility. However, 3 papers were excluded, one was an epidemiological study without enough details of the patient [9], one was only carotid cavernous fistula induced by PS deficiency [10], another one was not published in English language [11]. Finally, 4 articles [12–15] that reported 5 cases diagnosed as coexistence of CVST and dural AVF associated with PS deficiency were identified. A flow chart of the method of article retrieval is presented in Fig. 1.

Clinical characteristics of each patient, including age, gender, clinical presentations, location and sequence of CVST and dural AVF, level of PS, therapy, and prognosis, were extracted from the included studies. They were reviewed and summarized.

## Results

### Case presentation

One case was found from HGH medical records listed with diagnoses of both CVST and dural AVF related with deficiency of PS.

### Our case

**Fig. 2:**
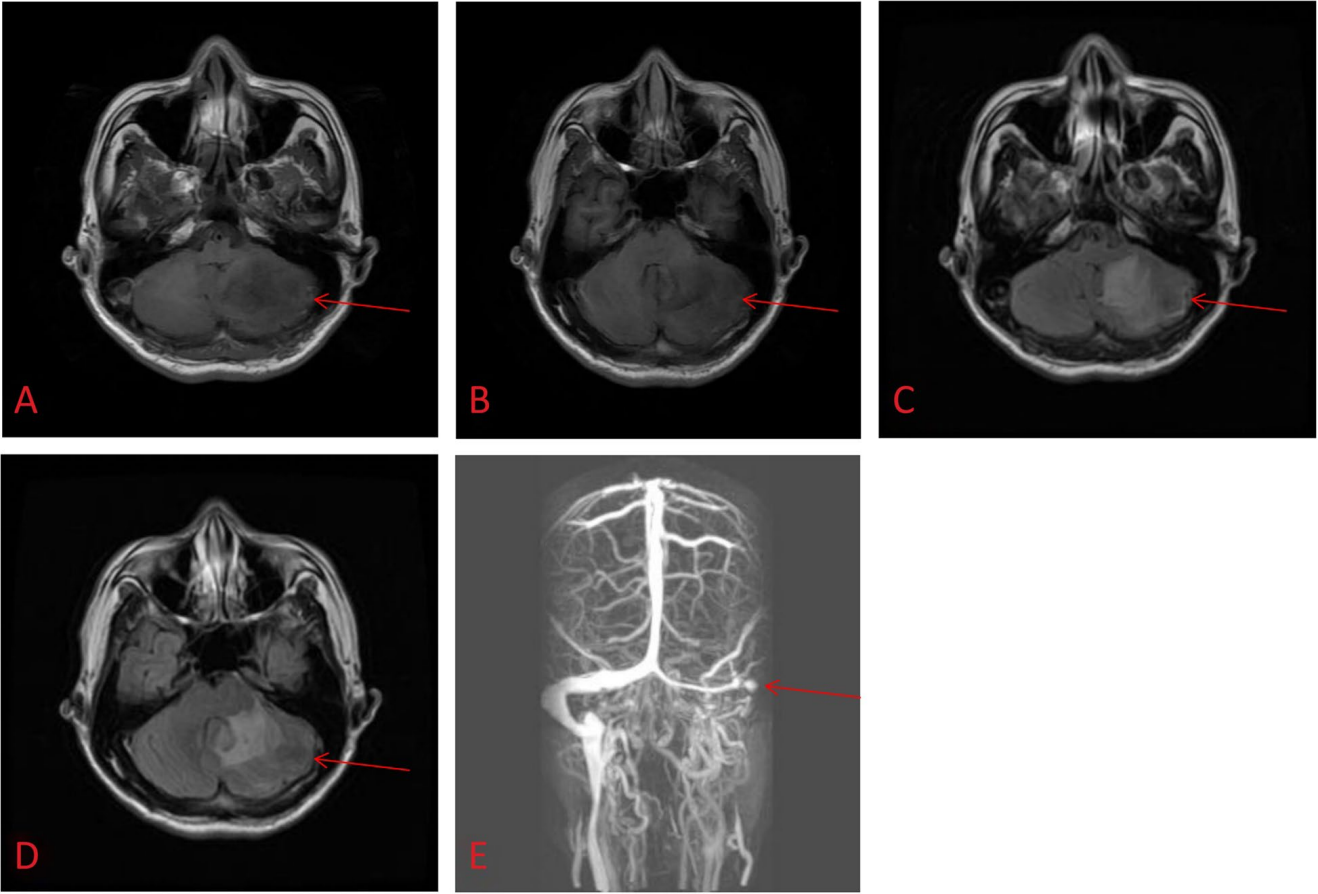
Cerebral MRI showed abnormal signal in left cerebellar hemisphere, vermis cerebelli, pontibrachium(**A**, **B**: T1W1 showed hypointensity, **C**: T2WI showed hyperintensity, **D**: Flair showed hyperintensity); MRV showed left sigmoid sinus was indistinct(**E**)

**Fig. 3:**
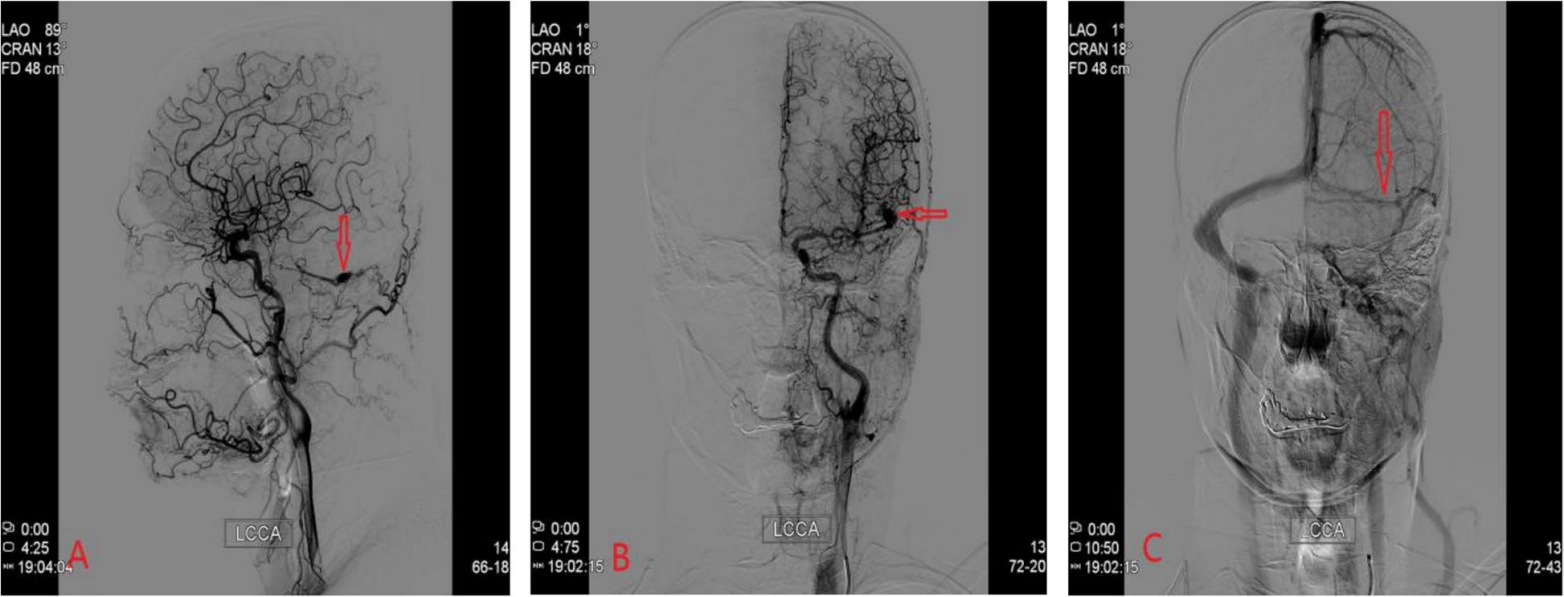
DSA revealed a dural AVF fed by the left occipital artery(**A**, **B**), occlusion of left sigmoid, torcular sinus and internal cerebral vein(**C**)

A 62-year-old man was admitted with headache and dizziness for 5 days. He also complained about intermittent nausea and vomiting. Physical examination was unremarkable. The patient’s medical and family history was normal and he did not take any medicine. The physical examination was normal. Hematologic values showed normal levels of protein C and antithrombin III. There was no factor 5 Leiden or antiphospholipid antibodies. The activity of PS decreased at 39.5%(normal:69–169%). There was no family history of venous thromboembolism or fetal wastage. Three days after admission a cerebral MRI was performed showing abnormal signal of left cerebellar hemisphere, vermis cerebelli, pontibrachium, and a suspicion of cerebral of thrombosis of left sigmoid sinus on venous MR angiogram (Fig. 2). Digital subtraction cerebral angiography (DSA) revealed occlusion of the deep venous system (left sigmoid, torcular sinus and internal cerebral vein), an asymptomatic dural AVF classified as Borden [16] Type II next to left transverse sinus was found, which was fed by the left occipital artery and drained into the sphenoparietal sinus with cortical reflux (Fig. 3). No embolization for the fistula was performed. The patient was treated with intravenous heparin and oral coagulation therapy was started at discharge. The patient showed a clinical improvement and 2 year follow-up showed no relapse after treatments.

### Clinical characteristics of CVST and dural AVF combined with PS deficiency

Altogether, the characteristics of 5 cases reported in the literature and 1 case from our own were reviewed.

The detailed clinical laboratory characteristics and treatments of 6 patients were shown in Tables 1, 2 and 3. The series was composed of 5(83.3%) men and 1(16.7%) woman. The mean age of patients was 41 ± 25.77 years (ranging from 1 to 71 years old). The most common symptoms were headache (3 patients, 50%), generalized seizure (3 patients, 50%), disturbance of consciousness (3 patients, 50%), other including nausea (2 patients, 33.3%), pulsatile tinnitus (2 patients, 33.3%), memory deficit (1 patient, 16.7%), muscle weakness (1 patient, 16.7%), dizziness (1 patient, 16.7%).

**Table 1:** Clinical characteristics of 6 cases

Author	Case	Gender	Age	Risk	Another thrombosis	Clinical symptoms	Treatment	Outcome
			(years)		event			
our case	1	M	62	no	no	headache,	anticoagulant	improved
						dizziness		
Mastubara [12]	2	M	38	no	DVT	headache,	anticoagulant,	improved
						generalized seizure	embolization	
	3	M	50	no	DVT	generalized seizure,	anticoagulant,	died
						pulsatile tinnitus	embolization	
Yassari [13]	4	F	24	surgery	no	pulsatile tinnitus	anticoagulant	improved
Witt [15]	5	M	1	infection	no	generalized seizure	anticoagulant,	died
							embolization	
Pasi [14]	6	M	71	no	DVT, PE	rapidly progressive	anticoagulant,	improved
						dementia	embolization	

*DVT* Deep venous thrombosis, *PE* Pulmonary embolism

**Table 2:** Level of protein S

Case	Deficiency of PS	Level of PS	Normal level of PS
1	reduced activity of PS	activity of PS:39.5%	60–150%
2	reduced antigen level and	tPS: 23%,	65–130%
	activity of tPS and fPS	fPS: 16%,	60–150%
		Activity of PS:10%	60–150%
3	reduced antigen level of fPS	fPS: 38%,	60–150%
		tPS: 112%	
4	reduced antigen level of tPS	tPS:62%	69–169%
5	reduced antigen level of	fPS: 40%(first time)	60–150%
	tPS and fPS	45%(second time)	60–150%
		tPS: 63%(first time)	
		52%(second time)	
6	no detailed information		

*PS* Protein S, *tPS* Total protein S, *fPS* Free protein S

**Table 3:** Location and relationship of CVST and dAVF

Case	CVST location	AVF location	Cortical venous drainage	Relationship of CVST and dAVF
1	Lt sigmoid, ICV, torcular	dAVF	yes	unclear
		SS		
2	SSS	dAVF+pial AVF	yes	CVST→dAVF
		Parietal		
3	SSS, Rt sigmoid	dAVF+pial AVF	yes	CVST→dAVF
		parietal, sigmoid		
4	Lt sigmoid, ICV	dAVF	no	unclear
		Lt TS		
5	Rt TS, Lt ICV	dAVF	no	unclear
		Torcular		
6	Lt TS, torcular, ICV, straight sinus, Galen vein	dAVF	no	unclear
		Lt TS, SSS,torcular		

*SSS* Superior sagittal sinus, *ICV* Internal cerebral vein, *Rt* Right, *Lt* Left, *TS* Transverse sinus, *SS*:Sphenoparietal sinus, *CVST* Cerebral venous sinus thrombosis, *dAVF* Dural arteriovenous fistula, *AVF* Arteriovenous fistula

One patient (case 2) showed low quantity and activity of fPS and tPS, one patient (case 3) presented with normal quantity of tPS and low quantity of fPS, one patient (case 4) revealed low quantity of tPS, one patient (case 5) displayed low quantity of fPS and low quantity of tPS, our case (case 1) revealed low activity of PS, one patient (case 6) did not supply detailed information about PS. Except for PS deficiency, two cases had other risk factors of thrombosis, one (case 2) was postoperative, another one (case 5) had upper respiratory infection. Three cases (case 2,3,6) had thrombotic events such as deep venous thrombosis and pulmonary embolism before.

The most frequent location of CVST was internal cerebral vein (4 patients, 66.7%), followed by the sigmoid sinus (3 patients, 50%), superior sagittal sinus (2 patients, 33.3%), transverse sinus (2 patients, 33.3%), torcula (2 patients, 33.3%), straight sinus (1 patient, 16.7%), Galen vein (1 patient, 16.7%). Two patients (case 2,3) presented with dural AVF and pial AVF, whereas other cases had dural AVF only. The location of dural AVF included: parietal region (2 patients, 33.3%), sigmoid sinus (2 patients, 33.3%), transverse sinus (2 patients, 33.3%), superior sagittal sinus (1 patient, 16.7%), sphenoparietal sinus (1 patient, 16.7%). Three patients (case 1,2,3) had cortical venous drainage. Two patients (case 2,3) had CVST first, and subsequent occurrence of dural AVF. Four patients (66.7%) were found to suffer from the two diseases at the same time, and it was difficult to distinguish the order of onset. All patients were treated with anticoagulant, four patients were treated with embolization, four patients recovered, two patients turned out to be dead.

## Discussion

Herein, we presented one case from our database and summarized the clinical characteristics of all the other cases that have been reported [12–15]. Coexistence of CVST and dural AVF is not rare, but associated with PS deficiency is infrequent. Clinical characteristics in coexistence of CVST and DAVF associated with PS deficiency are not specific, headache, generalized seizure and disturbance of consciousness were the most frequent symptoms, following by nausea and pulsatile tinnitus.

Dural AVF is a kind of abnormal arteriovenous anastomosis, belonging to the category of intracranial vascular malformation, which is clinically rare and can easily be misdiagnosed or never diagnosed [17]. Multiple studies have found thrombophilia to be highly prevalent among patients with dural AVF [18, 19]. The abnormalities stay between arteries of dura mater and veins of dura mater, cerebral venous sinus or cortical veins, accounting for about 10–15% of intracranial vascular malformation [20]. Besides, the abnormalities can occur at any part of the dura mater, but it is more likely at cavernous sinus, transverse sinus, sigmoid sinus and superior sagittal sinus [21]. In our study, sigmoid, transverse sinus and parietal region were more common. The pathogenesis of dural AVF is still unclear, which may be related to CVST, trauma, inflammation, level of estrogen, etc. . Inflammation or thrombosis of venous sinus caused by various factors lead to the obstruction of venous reflux, which gives rise to high pressure in the local venous drainage area, and then arteriovenous shunt opening, vascular remodeling and expansion of communicating branch in arteriovenous can result in dural AVF and nervous functional disorder [22]. CVST has a close relationship with dural AVF, 39–78% dural AVF patients suffer from CVST at the same time [23]. Pierot [24] reported that 3 out of 5 cases got CVST first and then suffered from dural AVF. A study showed that 5 patients had been cut off sigmoid and transverse sinus due to tumor operation, and all of them got dural AVF 2–6 years later [25]. Another research confirmed that hypertension of venous sinus in rats could increase the occurrence rate of new fistula, especially when it was combined with superior sagittal sinus thrombosis [26]. Therefore, CVST might become the risk factor for dural AVF through increasing venous sinus pressure. On the other hand, dural AVF could also result in CVST, the causal relationship between them is still controversial. Two patients in our case series had CVST first, and several years later developed dural AVF combined with pial AVF.

One patient in our report developed CVST and dural AVF several months after a tumor surgery, one patient was diagnosed with CVST and dural AVF several days after an upper respiratory tract infection. Our research indicated maybe a transient increase in acute proteins associated with surgery or upper respiratory tract infection could have promoted a pro-thrombogenic state, resulting in sinus thrombosis and subsequent formation of a dural AVF.

Hanaoka [27] reported a patient with dural AVF, whose fistula blocked by itself a week later surprisedly. It is speculated that AVF resulting in CVST would further led to blocking of the fistula [24]. Anticoagulant is the preferred treatment, however endovascular treatment for fistula could vary from patient to patient. Reasonable surgical intervention should be adopted to block fistulas while dural AVF have caused corresponding clinical symptoms or CVST. However, those dural AVF patients whose symptoms were minor (except trauma) or symptoms alleviated voluntarily, instant surgery was not recommended as fistula could block without any surgical intervention. All patients in our case series were treated with anticoagulant, four patients were treated with embolization in view of the symptoms caused by fistulas, one patient developed new fistula after the surgery. Two patients did not undergo surgery, several years follow-up showed no relapse.

PS, a major natural anticoagulant generated by liver cells, not only can be used as cofactor to activate protein C and promote inactivation of clotting factor like Va and VIIa which participates in anticoagulation but also can directly inhibit the formation of prothrombin protease complexes [28]. Superficial, intracranial, visceral or popliteal venous thrombosis caused by PS deficiency is relatively rare. PS deficiency can either be hereditary or acquired. Acquired PS deficiency can occur with oral contraceptive use, pregnancy, estrogen therapy, liver disease, and acute inflammatory processes [7]. Hereditary PS deficiency is autosomal dominant in inheritance, which is caused by multiple mutations involving the PROS1 gene [29, 30], with heterozygous mutations resulting in mild disease and homozygous mutations causing severe disease [31]. Hereditary PS deficiency is divided into three types based on PS antigen and activity levels. Type I has low total and free antigen levels with reduced activity, type II has normal total and free antigen levels with reduced activity, and type III has normal total antigen, reduced free antigen, and reduced activity. Clinically 95% of PS proficiency belongs to typeIorIII [32]. We consider all patients in our case series as hereditary PS deficiency for no risk factor of acquired PS deficiency was found. One case (his father had a reduced level of PS) reported suspicious related family history with PS deficiency. Three patients belonged to type I, one patient may belong to type I or III, two patients could not be clearly classified to any type.

All in all, based on the results of the case-series reported, PS deficiency is a major cause for hypercoagulability, which will further result in CVST combined with dural AVF. However, there are some limitations of our study. Firstly, since dural AVF might be a long-term complication of CVST, there may be an underestimation of its prevalence as it could be asymptomatic and go un-noticed if follow-up imaging is not performed. So after occurrence of CVST, attention should be paid by follow-up determine if dural AVF is accompanied. Moreover, given the limited case reports in our study, more evidences are needed in the future to further confirm the relationship between PS deficiency, CVST and dural AVF.

## Conclusion

Apart from CVST, the possibility of dural AVF should also be considered for patients who suffered from headache, seizure, disturbance of consciousness. Especially when those diagnosed with CVST don’t respond well to anticoagulant therapy or abnormal vascular flow emptyness on imaging or the condition worsens during the course of improvement or pulsating tinnnitus observed, a dural AVF should be screened through DSA. In addition, we should not ignore the PS deficiency in patients who suffer from CVST, particularly those combined with dural AVF. We conclude that the presentation of an unexplained cerebral thrombosis and arteriovenous malformation must alert the physician to screen for a possible inherited coagulation inhibitor deficiency, especially PS deficiency.

### Ethics statement

This study was approved by the Ethics Committee of Clinical Research of Hainan general hospital (Hainan, China), with written informed consent from the subject whose medical record was reviewed.

## Data Availability

All data generated or analysed during this study are included in this published article.
